# Association of coagulation dysfunction with cardiac injury among hospitalized patients with COVID-19

**DOI:** 10.1038/s41598-021-83822-9

**Published:** 2021-02-24

**Authors:** Liang Chen, Wei Hu, Xiaoxiao Guo, Ping Zhao, Jia Tang, Yuwei Gu, Ninghao Huang, Chao Wang, An Cui, Dian Zhang, Linjie Hu, Yi Feng, Shengshou Hu, Mingquan Chen, Firat Duru, Chenglong Xiong

**Affiliations:** 1 State Key Laboratory of Cardiovascular Disease, Fuwai Hospital, National Center for Cardiovascular Diseases, Chinese Academy of Medical Sciences and Peking Union Medical College, Beijing, China; 2 Department of Emergency, Huashan Hospital, Fudan University, 12 Middle Urumqi Road, Shanghai, 200040 China; 3 Department of Epidemiology, School of Public Health, Fudan University, 130 Dong’an Road, Shanghai, 200032 China; 4 Department of Integrative Medicine and Neurobiology, School of Basic Medical Sciences, Fudan University, Shanghai, China; 5 Department of Cardiology, University Heart Center, Raemistrasse 100, 8091 Zurich, Switzerland; 6 Center for Integrative Human Physiology, University of Zurich, Zurich, Switzerland; 7 Key Laboratory of Public Health Safety, Ministry of Education, School of Public Health, Fudan University, Shanghai, China

**Keywords:** Cardiovascular biology, Clinical microbiology

## Abstract

Cardiac injury is a common complication of the coronavirus disease 2019 (COVID-19), and is associated with adverse clinical outcomes. In this study, we aimed to reveal the association of cardiac injury with coagulation dysfunction. We enrolled 181 consecutive patients who were hospitalized with COVID-19, and studied the clinical characteristics and outcome of these patients. Cardiac biomarkers high-sensitivity troponin I (hs-cTnI), myohemoglobin and creatine kinase-myocardial band (CK-MB) were assessed in all patients. The clinical outcomes were defined as hospital discharge or death. The median age of the study cohort was 55 (IQR, 46–65) years, and 102 (56.4%) were males. Forty-two of the 181 patients (23.2%) had cardiac injury. Old age, high leukocyte count, and high levels of aspartate transaminase (AST), D-dimer and serum ferritin were significantly associated with cardiac injury. Multivariate regression analysis revealed old age and elevated D-dimer levels as being strong risk predictors of in-hospital mortality. Interleukin 6 (IL6) levels were comparable in patients with or without cardiac injury. Serial observations of coagulation parameters demonstrated highly synchronous alterations of D-dimer along with progression to cardiac injury. Cardiac injury is a common complication of COVID-19 and is an independent risk factor for in-hospital mortality. Old age, high leukocyte count, and high levels of AST, D-dimer and serum ferritin are significantly associated with cardiac injury, whereas IL6 are not. Therefore, the pathogenesis of cardiac injury in COVID-19 may be primarily due to coagulation dysfunction along with microvascular injury.

## Introduction

The pandemic of coronavirus disease 2019 (COVID-19), which is caused by severe acute respiratory syndrome coronavirus 2 (SARS-CoV-2)^1^, has resulted in considerable morbidity and mortality in millions of infected patients. Many studies have already described the clinical characteristics of patients with COVID-19^2–5^. The overall mortality of the COVID-19 was determined to be 2.3% among general laboratory-confirmed cases^6^, but the fatality rate was more than 10% among hospitalized patients, and even reached to 40% among critically ill cases^5^. Cardiac injury, manifesting as elevation of troponin I (TnI) and decline of left ventricular ejection fraction, has been observed as a common complication in 10–30% of hospitalized cases, significantly increasing the severity of COVID-19. It was also demonstrated to be an independent risk factor for mortality^5,7,8^. Thus, understanding cardiac involvement and its monitoring in the clinical setting are of utmost importance for risk stratification and management of infected patients.

The pathogenic mechanisms underlying cardiac injury in COVID-19 remain unclear. Multiple hypotheses have been proposed to be potentially associated with cardiac injury, such as myocarditis directly induced by the virus through angiotensin-converting enzyme 2 (ACE2)^9,10^, the putative viral receptor of SARS-COV-2^11^. Other proposed mechanisms include cardiomyocyte damage induced by inflammatory cytokines or hypoxemia due to respiratory dysfunction^12^ but the association of cardiac injury with these factors has not been systematically illuminated. In this study, we aimed to reveal the mechanisms of cardiac involvement and its association with coagulation dysfunction, and identify the potential risk factors for cardiac injury in hospitalized patients with COVID-19.

## Results

### Patient characteristics and laboratory findings

Data from 181 consecutive patients hospitalized with COVID-19 were enrolled in this study, including 42 patients (23.2%) with cardiac injury and 139 patients (76.8%) without cardiac injury (Table 1). The median age of the patients in the overall cohort was 55 (IQR, 46–65) years, and 102 (56.4%) were males. There were more males in patients with cardiac injury, and these patients were older and had baseline comorbidities, such as hypertension, coronary heart disease, arrhythmias and cancer, more often.

**Table 1:** Clinical characteristics of patients with or without cardiac injury.

Characteristics	Total (n = 181)	Non-cardiac injury (n = 139)	Cardiac injury (n = 42)	*P* value
Age, years	55 (46, 65)	54 (43, 64)	63.5 (50, 71)	0.002
Male	102 (56.4)	71 (51.1)	31 (73.8)	0.009
Heart rate	88 (80, 96)	87 (80, 95)	89.5 (82, 96)	0.142
Systolic pressure, mmHg	128 (116, 142)	128 (116, 140)	133.5 (119, 145)	0.385
Diastolic pressure, mmHg	80 (72, 85)	80 (71, 85)	82 (72, 86)	0.793
Smoking	15 (8.3)	9 (6.5)	6 (14.3)	0.118
Comorbidities, n(%)				
Hypertension	54 (29.8)	37 (26.6)	17 (40.5)	0.086
Diabetes	29 (16)	21 (15.1)	8 (19)	0.543
Coronary heart disease	8 (4.4)	4 (2.9)	4 (9.5)	0.086
Arrhythmias	5 (2.8)	2 (1.4)	3 (7.1)	0.083
COPD	8 (4.4)	4 (2.9)	4 (9.5)	0.086
Cancer	7 (3.9)	3 (2.2)	4 (9.5)	0.052
Infective disease	10 (5.5)	7 (5)	3 (7.1)	0.700
Lab findings, median (IQR)				
SpO_2_ (%)	0.96 (0.9, 0.98)	0.97 (0.93, 0.98)	0.89 (0.77, 0.94)	< 0.001
Leukocytes, × 10^3^/μL	5.61 (4.08, 8.48)	5.27 (3.9, 7.33)	8.35 (5.02, 11.26)	0.001
Erythrocytes, × 10^6^/μL	4.19 (3.86, 4.56)	4.19 (3.88, 4.53)	4.12 (3.84, 4.58)	0.922
Hemoglobin, g/dL	129 (117, 136)	128 (118, 136)	129.5 (116, 136)	0.810
Platelets, × 10^3^/μL	191 (147, 251)	203 (153, 269)	152 (125, 208)	0.002
Lymphocytes, × 10^3^/μL	0.98 (0.67, 1.61)	1.17 (0.8, 1.72)	0.76 (0.48, 0.95)	< 0.001
Neutrophils, × 10^3^/μL	5.81 (2.64, 7.49)	4.72 (2.43, 5.43)	9.4 (4.14, 11.48)	< 0.001
Monocytes, × 10^3^/μL	0.39 (0.28, 0.47)	0.39 (0.28, 0.49)	0.4 (0.21, 0.45)	0.160
Basophils, × 10^3^/μL	0.02 (0.01, 0.02)	0.02 (0.01, 0.02)	0.03 (0, 0.02)	0.453
Eosnophils, × 10^3^/μL	0.04 (0, 0.06)	0.05 (0, 0.07)	0.02 (0, 0.02)	< 0.001
Creatinine, μmol/L	68.4 (56.7, 81.7)	67 (56.7, 78.2)	73.6 (61, 93)	0.061
eGFR, × mL/(min × 1.73 m^2^)	106.13 (91.02, 127.69)	110.31 (95.06, 127.69)	102.12 (83.37, 129.5)	0.165
Albumin, g/L	33.1 (29.2, 36.5)	33.4 (30.4, 37.3)	30.6 (27.1, 33.5)	< 0.001
Total bilirubin, μmol/L	12.6 (9.4, 15.9)	12.4 (9.3, 15.5)	13.55 (9.6, 19.1)	0.207
AST, U/L	34 (25, 45)	32 (24, 41)	45 (32, 58)	< 0.001
ALT, U/L	33 (22, 46)	31 (20, 45)	37 (25, 54)	0.129
Cholesterol, mmol/L	3.88 (3.19, 4.53)	3.95 (3.22, 4.53)	3.69 (3.12, 4.62)	0.222
Triglyceride, mmol/L	1.2 (0.93, 1.76)	1.2 (0.9, 1.76)	1.21 (0.97, 2.16)	0.661
LDL-C, mmol/L	2.21 (1.74, 2.65)	2.22 (1.76, 2.68)	2.21 (1.55, 2.53)	0.351
HDL-C, mmol/L	1.03 (0.82, 1.23)	1.06 (0.86, 1.23)	0.9 (0.77, 1.24)	0.075
LDH,U/L	352 (236, 473)	244 (200, 346)	443.5 (318, 709)	< 0.001
hs-CRP, mg/L*	25.9 (6.2, 86.7)	15.1 (4.2, 66.4)	67.75 (25.4, 160)	< 0.001
Procalcitonin, ng/mL	0.05 (0.05, 0.1)	0.05 (0.05, 0.05)	0.11 (0.05, 0.25)	< 0.001
D-dimer, μg/mL	0.73 (0.36, 2.95)	0.58 (0.3, 1.21)	5.86 (0.81, 28.89)	< 0.001
Serum ferritin, ng/mL^†^	609.88 (304.31, 1217.78)	459.57 (262.78, 813.17)	1375.8 (967.81, 2000)	< 0.001
ESR, mm/h	42.05 (27.3, 63)	40 (27, 61)	48.2 (38.5, 66)	0.054
Interleukin 6, pg/mL	8.66 (6.65, 12.17)	8.36 (6.37, 12.12)	10.41 (6.79, 15.21)	0.089
hs-TnI, pg/mL	3.9 (1.2, 12.5)	2.9 (1, 6.9)	19.35 (9.4, 185.3)	< 0.001
Myohemoglobin, ng/mL	46.8 (28.2, 88.25)	37.9 (26.2, 65.2)	157.65 (120.4, 238)	< 0.001
BNP, pg/mL	31.1 (10.03, 77.37)	31.1 (10, 66.2)	59.3 (26.7, 256.7)	0.001
Creatine kinase, U/L	84 (55, 163)	76 (49, 122)	238 (97, 384)	< 0.001
CK-MB, U/L	14 (10, 18)	13 (10, 16)	18 (14, 34)	< 0.001

ALT: alanine aminotransferase; AST: aspartate transaminase; BNP: B-type natriuretic peptide; CK-MB: creatine kinase-myocardial band; COPD: chronic obstructive pulmonary disease; eGFR: estimated glomerular filtration rate; ESR: erythrocyte sedimentation rate; HDL-C: high density lipoprotein cholesterol; hs-CRP: high-sensitivity C-reactive protein; LDH: Lactic dehydrogenase; LDL-C: low density lipoprotein cholesterol.

*Extra levels of hs-CRP were shown as > 160; ^†^extra levels of serum ferritin shown as > 2000.

The duration from the beginning of symptoms to hospital admission was similar in patients with and without cardiac injury (median [IQR] days, 10 [7, 15] vs 10 [7, 15], *P* = 0.925). In addition to significantly elevated hs-TnI, myohemoglobin and CK-MB, patients with cardiac injury showed higher leukocyte count and higher levels of aspartate transaminase, high-sensitivity C-reactive protein (hs-CRP), D-dimer, procalcitonin, serum ferritin and lactic dehydrogenase (LDH), and lower levels of SpO2, platelets, lymphocytes, and albumin. All these findings were consistent with deteriorated conditions of the patients. Serum creatinine levels, lipids, erythrocyte sedimentation rate (ESR), and Interleukin 6 (IL6) (8.36 [6.37, 12.12] vs 10.41 [6.79, 15.21], *P* = 0.089) were comparable between the two groups.

### Clinical course and outcomes

**Figure 1:**
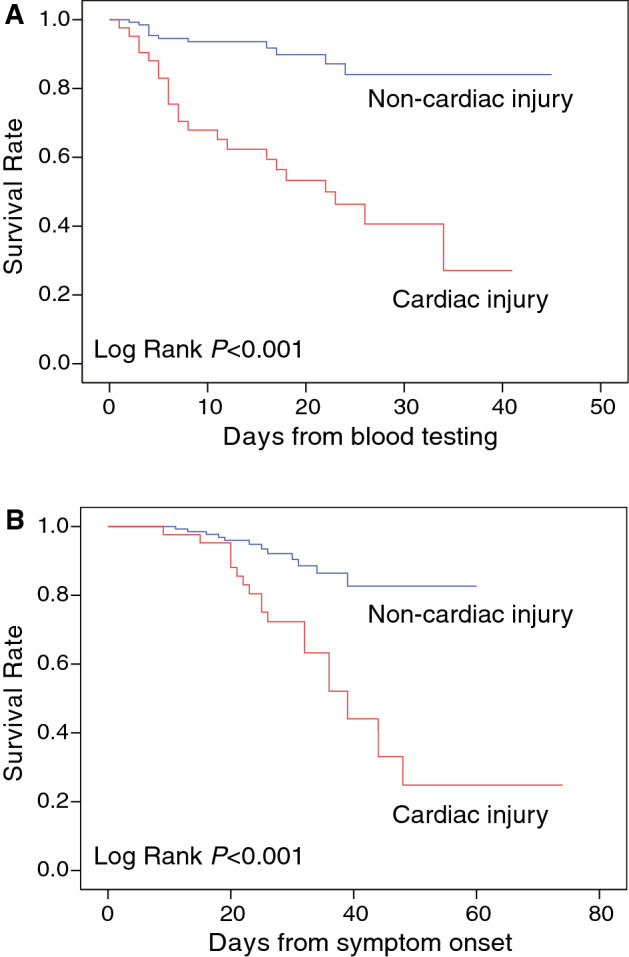
Kaplan–Meier curve. Kaplan–Meier curve for mortality of patients with or without cardiac injury during the time from blood testing (**A**) and symptom onset (**B**).

The treatment, complications and outcomes of the COVID-19 patients are shown in Table 2. The majority of patients were treated with anti-viral medications (95.0%), antibiotics (96.1%) and oxygen inhalation (74.6%). The patients in the cardiac injury group more often required glucocorticoids (29 [69.0%] vs 44 [31.7%], *P* < 0.001), immunoglobulin therapy (18 [42.9%] vs 26 [18.7%], *P* = 0.001) and noninvasive ventilation (20 [47.6%] vs 14 [10.1%], *P* < 0.001). Moreover, cardiac injury group had significantly more frequent hypoproteinemia (22 [52.4%] vs 33 [23.7%], *P* < 0.001) and ARDS (14 [33.3%] vs 12 [8.6%], *P* < 0.001), and more often progressed to severe disease (36 [85.7%] vs 47 [33.8%], *P* < 0.001). As a coincident, the mortality rate was higher in the cardiac injury group as compared to non-cardiac injury group (22 [52.4%] vs 12 [8.6%], *P* < 0.001). Kaplan–Meier survival curves are shown in Fig. 1 (Log rank, *P* < 0.001).

**Table 2:** Treatment, complications and clinical outcome of patients with COVID-19.

Characteristics	Total (n = 142)	Non-cardiac injury (n = 139)	Cardiac injury (n = 42)	*P* value
Time from onset to admission	10 (7.15)	10 (7.15)	10 (7.15)	1.000
Treatment				
Antiviral drugs	172 (95)	132 (95)	40 (95.2)	1.000
Antibiotics	174 (96.1)	133 (95.7)	41 (97.6)	1.000
Glucocorticoids	73 (40.3)	44 (31.7)	29 (69)	< 0.001
Immunoglobulin therapy	44 (24.3)	26 (18.7)	18 (42.9)	0.001
Oxygen inhalation	135 (74.6)	102 (73.4)	33 (78.6)	0.500
Noninvasive ventilation	34 (18.8)	14 (10.1)	20 (47.6)	< 0.001
Invasive ventilation	2 (1.1)	0 (0)	2 (4.8)	0.053
Complications				
ARDS	26 (14.4)	12 (8.6)	14 (33.3)	< 0.001
Acute kidney injury	8 (4.4)	4 (2.9)	4 (9.5)	0.086
Hypoproteinemia	55 (30.4)	33 (23.7)	22 (52.4)	< 0.001
Anemia	16 (8.8)	12 (8.6)	4 (9.5)	0.767
Hypoxemia	55 (30.4)	37 (26.6)	18 (42.9)	0.046
Arrhythmias	11 (6.1)	5 (3.6)	6 (14.3)	0.02
Clinical outcome				
Critical condition	83 (45.9)	47 (33.8)	36 (85.7)	< 0.001
Death	34 (18.8)	12(8.6)	22 (52.4)	< 0.001

### Predictors of in-hospital outcome

**Figure 2:**
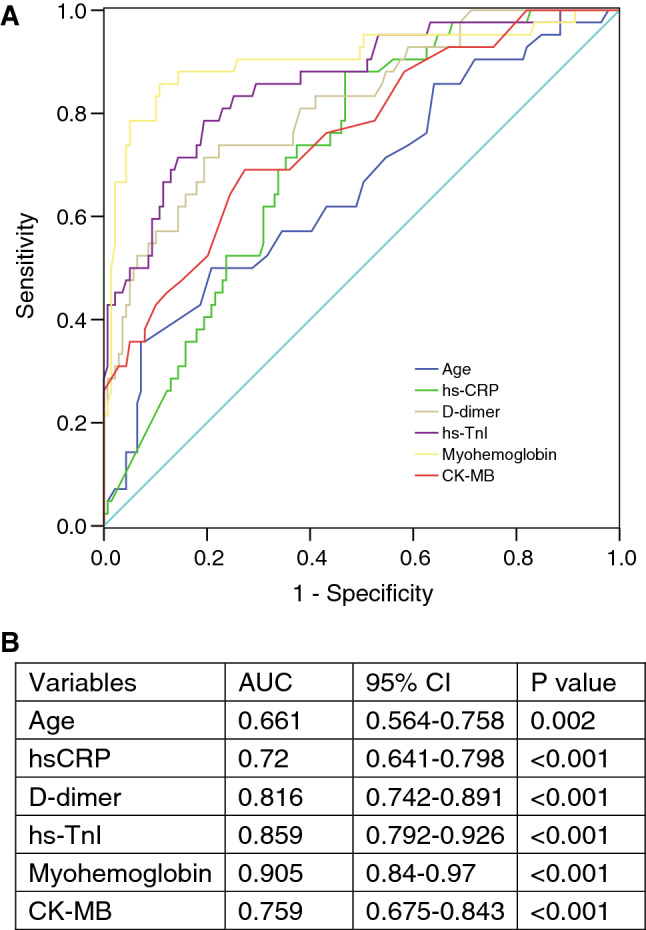
Receiver operating characteristic (ROC) analysis of the prediction of in-hospital mortality. (**A**) ROC curve of age and laboratory biomarkers. (**B**) Area under the ROC curve (AUC) with 95% confidence interval (CI) of each variable. CK-MB, creatine kinase-myocardial band; hs-TnI, high sensitivity-cardiac troponin I; hs-CRP, high-sensitivity C-reactive protein.

To examine the role of cardiac injury for the risk stratification of in-hospital COVID-19 mortality, we systematically compared the clinical characteristics between survivors (n = 147) and non-survivors (n = 34) (Table S1). Multivariable Cox regression analysis revealed that two of the three cardiac biomarkers was independently associated with the prognosis, with hazard ratios [95%CI] for myohemoglobin, and CK-MB being 1.41 ([1.11, 1.80], *P* = 0.005), and 1.50 ([1.10, 2.06], *P* = 0.012), respectively (Table 3). When we used binary variable as cardiac injury defined by elevation of cardiac biomarkers, it was also an independent predictor for mortality (2.63 [1.38, 5.65], *P* = 0.016). Other predictors such as age, hs-CRP, and D-dimer were also associated with poor prognosis in at least one model. ROC curve analysis demonstrated that these variables, and especially D-dimer and myohemoglobin, could independently well predict adverse outcomes (Fig. 2).

**Table 3:** Multivariate Cox regression analysis on the risk factors associated with mortality in patients.

Variables	Model 1		Model 2		Model 3		Model 4	
	HR (95%CI)	*P* value	HR (95%CI)	*P* value	HR (95%CI)	*P* value	HR (95%CI)	*P* value
Age	1.03 (1.01, 1.06)	0.016		0.083		0.014		0.058
COPD		0.053		0.093		0.030		0.063
SpO2, %		0.391		0.330		0.276		0.391
Leukocytes, × 10^3^/μL		0.696		0.903		0.770		0.696
Platelets, × 10^3^/μL		0.215		0.103		0.070		0.215
Lymphocytes, × 10^3^/μL		0.304		0.464		0.439		0.304
AST, U/L		0.186		0.937		0.386		0.186
LDH,U/L		0.401		0.758		0.322		0.401
hs-CRP, mg/L	1.44 (1.12, 1.85)	0.004	1.39 (1.08, 1.79)	0.011	1.43 (1.12, 1.82)	0.004	1.44 (1.12, 1.85)	0.004
D-dimer, μg/mL	1.47 (1.28, 1.71)	< 0.001	1.36 (1.18, 1.58)	< 0.001	1.46 (1.26, 1.68)	< 0.001	1.48 (1.28, 1.71)	< 0.001
Serum ferritin, ng/mL		0.055		0.330		0.321		0.055
ESR, mm/h		0.288		0.115		0.420		0.288
Interleukin 6, pg/mL		0.192		0.150		0.132		0.192
hs-TnI, pg/mL		0.218		–		–		–
Myohemoglobin, ng/mL		–	1.41 (1.11, 1.80)	0.005		–		–
CK-MB, U/L		–		–	1.50 (1.10, 2.06)	0.012		–
Cardiac injury		–		–		–	2.63 (1.38, 5.65)	0.016

Cardiac biomarkers were included in Model 1, 2, 3 separately as continuous variables. Cardiac injury was included in Model 4 as binary variable.

### Predictors of cardiac injury

As a next step, we performed Spearman correlation and revealed the potential links between cardiac injury biomarkers and other clinical characteristics such as SpO2, leukocytes, platelets, lymphocytes, aspartate transaminase (AST), LDH, hs-CRP, procalcitonin, D-dimer, serum ferritin and ESR (Table 4). However, the associations between cytokine IL6 and cardiac biomarkers were limited (*P* = 0.01 with hs-TnI, *P* = 0.346 with myohemoglobin, and *P* = 0.226 with CK-MB).

**Table 4:** Spearman correlation analysis between cardiac biomarkers and other laboratory findings.

Variable	hsTnI		Myohemoglobin		CK-MB	
	Spearman	*P* value	Spearman	*P* value	Spearman	*P* value
Age	0.47	< 0.001	0.40	< 0.001	− 0.03	0.693
SpO2	− 0.48	< 0.001	− 0.45	< 0.001	− 0.23	0.002
Leukocytes	0.28	< 0.001	0.21	0.005	0.09	0.236
Platelets	− 0.23	0.002	− 0.27	< 0.001	− 0.24	0.001
Lymphocytes	− 0.26	< 0.001	− 0.31	< 0.001	− 0.29	< 0.001
AST	0.28	< 0.001	0.35	< 0.001	0.44	< 0.001
LDH	0.41	< 0.001	0.46	< 0.001	0.57	< 0.001
hs-CRP	0.45	< 0.001	0.42	< 0.001	0.28	< 0.001
PCT	0.42	< 0.001	0.39	< 0.001	0.37	< 0.001
D-dimer	0.47	< 0.001	0.40	< 0.001	0.15	0.035
Serum ferritin	0.50	< 0.001	0.57	< 0.001	0.35	< 0.001
ESR	0.21	0.004	0.15	0.04	0.15	0.042
IL6	0.19	0.010	0.07	0.346	− 0.09	0.226

**Figure 3:**
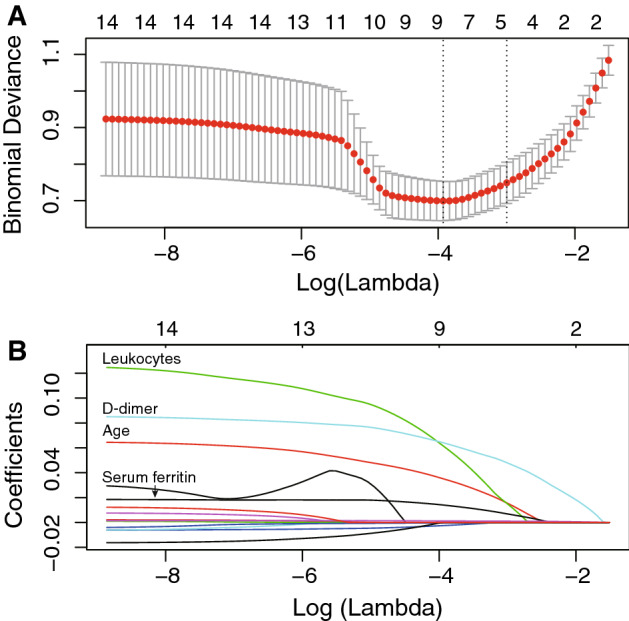
Feature selection using the LASSO binary regression model to identify independent factors of cardiac injury. (**A**) Tuning parameter (λ) selection in the LASSO model used tenfold cross-validation via minimum criteria. The binomial deviance was plotted versus log (λ). Dotted vertical lines were drawn at the optimal values by using minimum criteria and the 1 standard error (1-SE criteria). (**B**) LASSO coefficient profiles of the 15 features. A coefficient profile plot was produced against the log(λ) sequence. Dotted vertical line was drawn at the optimal λ at minimum criteria and 1 standard error (1-SE criteria). The model at 1-SE criteria was selected as the final model with 4 nonzero coefficients including age, leukocytes, D-dimer, and serum ferritin. LASSO, least absolute shrinkage and selection operator.

**Figure 4:**
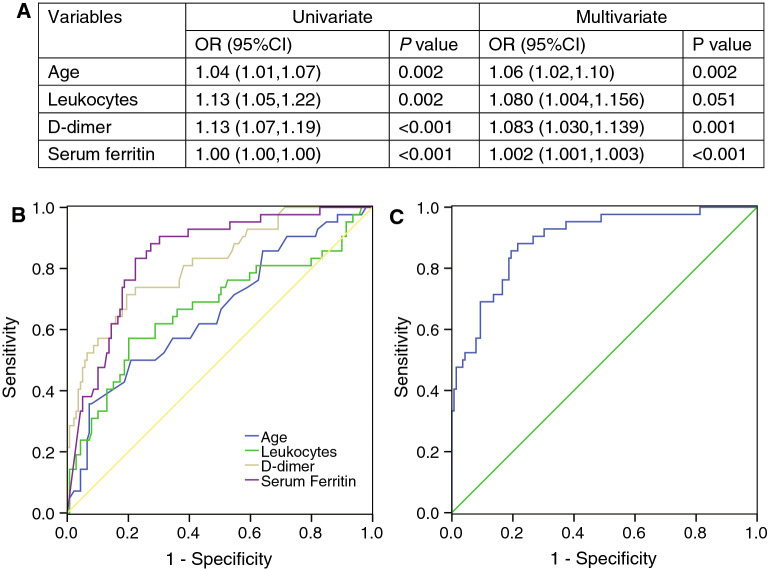
Receiver operating characteristic (ROC) analysis of the prediction of cardiac injury by multivariate regression model. (**A**) Univariate and multivariate logistic regression analysis of cardiac injury. (**B**) ROC curve of the prediction of cardiac injury by each variable. (**C**) ROC curve of the prediction of cardiac injury by the combined regression model of five variables. Area under the ROC curve (AUC) was 0.896, with a sensitivity of 83.30% and specificity of 85.30%.

To further examine the predictors of cardiac injury, we used two multivariable regression models, LASSO and logistic regression. In LASSO regression analysis, we identified four features with nonzero coefficients including old age, leukocytes, D-dimer and serum ferritin from a total of 15 related variables (Fig. 3). Logistic regression analysis also confirmed exactly the same four independent predictors of cardiac injury in patients with COVID-19 (Table S2, Fig. 4A). Each factor showed good discrimination of cardiac injury through ROC curve analysis (*P* < 0.001 in each factor), especially serum ferritin (AUC, 0.844) and D-dimer (AUC, 0.816) (Fig. 4B). We established a logistic regression model based on the combination of the four predictors, and the ROC curve showed excellent discrimination of cardiac injury (AUC, 0.896) with a sensitivity of 83.30% and specificity of 85.30% (Fig. 4C).

### Cardiac injury and coagulation

**Figure 5:**
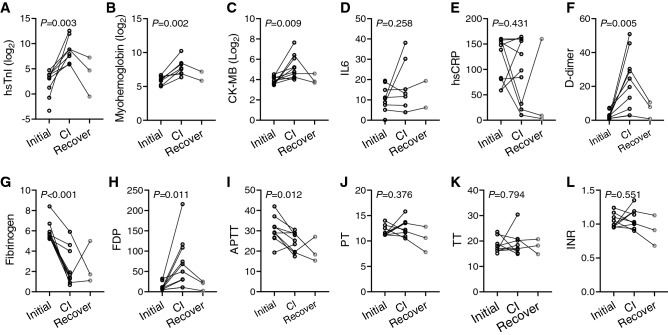
Dynamic changes of cardiac, inflammatory and coagulation biomarkers. The laboratory testing results were obtained from initial time on admission, to cardiac injury (CI) state manifested by elevation of cardiac biomarkers. The recovery state from three patients were also included. *P* value represented comparison of initial and cardiac injury state, by paired Student’s *t*-test. FDP, fibrinogen degradation products; aPTT, activated partial thromboplastin time; PT, Prothrombin time; TT, thrombin time; INR, international normalized ratio.

**Figure 6:**
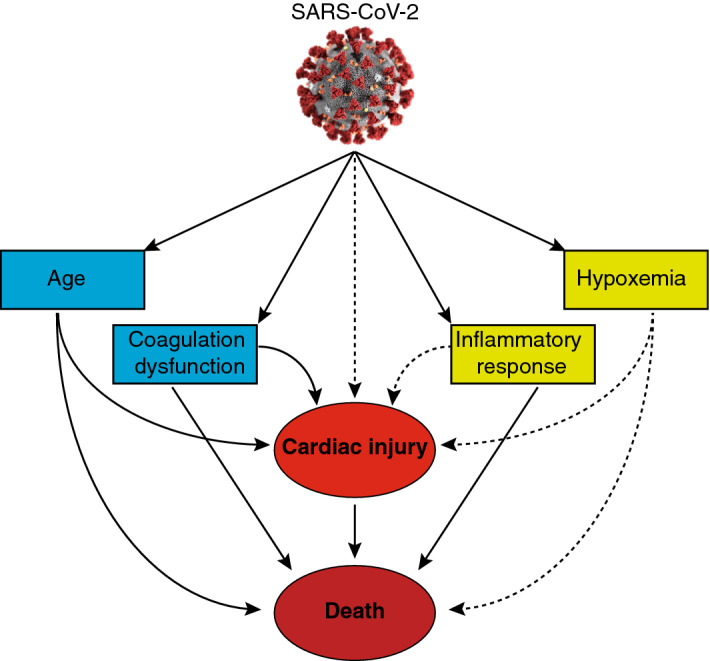
Work Summary. The patients with COVID-19 could have multiple manifestations like elder age, coagulation dysfunction, cardiac injury, inflammatory and hypoxemia, of which the former four factors are associated with in-hospital mortality. In the meantime, cardiac injury is associated with senior age and coagulation, but not directly associated with inflammatory response and hypoxemia.

Considering the significant association of D-dimer with cardiac injury, we further validated the role of coagulation dysfunction in the development of cardiac injury. We involved a sub-cohort containing 10 patients who were free of cardiac involvement at admission, but developed cardiac injury during later hospitalization. Serial testing of biomarkers in these patients revealed consistent elevation of cardiac biomarkers, and three of them alleviated during follow-up (Fig. 5A–C). The inflammatory biomarkers IL6 and hs-CRP were generally elevated among these patients, but no synchronous change (elevation or decrease) was observed consistent with cardiac injury (Fig. 5D,E). On the other hand, markers of coagulation function, D-dimer (*P* = 0.005) and fibrinogen degradation products (FDP) (*P* = 0.011), were significantly increased from the time of admission to the time of cardiac injury, while fibrinogen (*P* < 0.001) and aPTT (*P* = 0.012) were synchronously decreased along with the progression of cardiac involvement (Fig. 5F–L). These results suggest that cardiac injury was closely accompanied by hypercoagulable state. In addition, the levels of D-dimer and FDP recovered back to initial state among the three patients with alleviated cardiac injury during follow-up, which were consistent with the dynamic alterations of cardiac biomarkers. The summary of discoveries from this study is shown in Fig. 6.

## Discussion

This study systematically analyzed the occurrence cardiac injury and described its clinical predictors in patients with COVID-19. The major findings of this study include: (i) cardiac injury, which is manifested by elevation of cardiac biomarkers, is an independent risk factor for in-hospital mortality; (ii) old age, high leukocyte count, D-dimer and serum ferritin are significantly associated with cardiac injury; and (iii) the dynamic progression of cardiac injury is particularly correlated with coagulation dysfunction (elevated D-dimer and FDP and decreased fibrinogen). While two of the above findings (i and ii) confirm the results of previously reported studies, the latter (iii) is a novel and most important finding of this study (Fig. 6).

### Cardiac injury associated with poor prognosis

The COVID-19 has widely spread since its first reported outbreak in December 2019 and has not been controlled to date. Previous studies have demonstrated that SARS-CoV-2 not only causes pneumonia, but also may result in multiple organ injury through systemic inflammation or due to direct attack of the virus via ACE2 receptor^3,5,6,13^. Cardiac injury has been reported to be common among COVID-19 patients, ranging from 7.2 to 27.8% of all hospitalized patients^7,8,14^, and its incidence has been higher among patients who suffered from severe disease requiring intensive care unit (ICU) or in those who died^5,15^. Pre-exiting comorbidities such as hypertension, coronary heart disease, and COPD have been identified as risk factors for cardiac injury^14^. However, it was also observed that 11.8% of patients who died from COVID-19 had no baseline underlying cardiovascular condition, and despite that, these patients developed substantial cardiac damage^12^. Interestingly, in our cohort, cardiac injury was more commonly observed in male patients than in females (*P* = 0.009), which could be partially explained by significantly higher rate of smokers among males in China, and by a recent observation that showed higher plasma concentration of ACE2 in men as compared to women^16^.

Consistent with previous studies^7,8,14^, our study confirmed that patients with cardiac injury had considerably higher in-hospital mortality rate (52.4%) than those who did not develop cardiac injury (8.6%). Moreover, several clinical parameters, such as old age, IL6 and coagulation parameters (especially D-dimer) have also been reported to be associated with adverse prognosis by retrospective cohort studies^17,18^. However, there was no reported evidence to reveal the role and weight of cardiac injury in predicting prognosis compared to the other laboratory parameters. In present study, we included multiple associated factors in the multivariate regression model, and demonstrated the independent role of cardiac injury, defined by binary variable or continuous variable (hs-TnI, myohemoglobin and CK-MB), in predicting the risk for death. Meanwhile, old age, D-dimer and hs-CRP levels were also independent predictors for poor prognosis, which was consistently reported by previous studies^17,19^. Due to high proportion of critically ill cases among patients with cardiac injury group, simultaneously with high leukocyte and hs-CRP, the immunosuppress medication such as glucocorticosteroids and immunoglobulin was widely used among these patients in this cohort at very early stage of COVID-19 outbreak.

These results emphasize the fundamental role of cardiac injury in deteriorating the clinical course of patients with COVID-19, and thus, close clinical monitoring and early intervention in case of cardiac injury are of utmost importance for management of these patients.

### Cardiac injury and inflammation

The pathogenic mechanisms of cardiac injury caused by SARS-CoV-2 remain controversial^20^. A recent study identified that old age, comorbidities and CRP were risk factors of cardiac injury in patients with severe COVID-19 by multivariate regression analysis^14^. However, most simultaneous blood testing results were not included in this model. In contrast, our study focused on the association of cardiac injury with other pathophysiological alterations such as renal and hepatic function, coagulation parameters, as well as inflammatory mediators, to reveal the potential mechanisms that may contribute to the cardiac injury development during the progression of disease.

The theory of inflammatory response inducing myocardial injury has been widely discussed including direct viral invasion of cardiomyocytes through ACE2 receptors and systemic inflammation (e.g. cytokine storm) inducing cardiomyocyte injury^10,20^. Serial cases studies reported myocardial involvement manifested as myocarditis-like presentation or Tako-Tsubo syndrome^21–23^. Likewise, cardiac infection by viral particles in endomyocardial biopsy was also observed by electron microscopy in a case report^24^. Thus, myocarditis caused by virus directly attacking cardiomyocytes was inferred in these cases. However, autopsy examination also revealed interstitial immune cell infiltrates without substantial myocardial damage in patients with COVID-19^25^. Therefore, it can be speculated that typical myocarditis-like presentation does exist in some patients, but this mechanism is likely not responsible for the considerable proportion of hospitalized cases having cardiac injury.

Higher blood levels of inflammatory biomarkers (e.g. CRP) and cytokines (e.g. IL2, IL7, IL10, etc.) have been reported in COVID-19 patients admitted to the ICU^3^, and IL6 and IL10 have been determined to be strong discriminators for severe disease and death, consistent with our findings^26,27^. These discoveries consistently indicated intense systemic inflammatory response in severe COVID-19 cases. However, a direct connection between systemic inflammatory markers and cardiac injury has not been established. In our present study, IL6 was generally increased among patients with COVID-19, and was further elevated in the non-survivor group compared to survivors, in accordance with a previous study^27^. On the other hand, IL6 was comparable between patients with or without cardiac injury in our study. Likewise, another inflammatory biomarker hs-CRP was not significantly associated with cardiac injury in our multivariable regression model. Serial testing analysis also revealed that the dynamics of IL6 and hs-CRP were not consistent with cardiac biomarker changes during progression of cardiac injury. It is noteworthy that few patients (2 out of 10 cases) presented dramatic increase of IL6 along with cardiac injury progression, suggesting that small proportion of patients might indeed have cytokine storm in this process. This may explain the observed association of plasma inflammatory cytokines and cardiac injury among critically ill patients as reported before^28^. Nevertheless, our result suggests that, in general, both systemic inflammation and cardiac injury are associated with disease severity and prognosis, but any direct correlations between systemic inflammation induced by viral infection and cardiac injury still remain to be elucidated. Nevertheless, the localized inflammation, distinctive from the systemic inflammation, is non-negligible in the pathogenesis of cardiac injury. The evaluation of localized inflammation is not available in present study, but it is widely confirmed in the pathogenesis of cardiac thrombosis and endothelial dysfunction.

### Cardiac injury and coagulation dysfunction

In this present study, old age, high leukocyte count and elevated levels of AST, serum ferritin and D-dimer were demonstrated to be independently associated with cardiac injury using different multivariable analyses. Of note, old age and D-dimer were also demonstrated to be strong risk predictors of in-hospital mortality using multivariate regression analysis^17,20^. Moreover, it was reported that discrete changes in D-dimer levels were observed earlier in the course of disease preceding the rapid progression stage^20^. In some patients, increased release of IL6 can also initiate coagulation activation and contribute to hypercoagulable state.

Serial blood testing analysis revealed that dynamic changes of D-dimer were highly consistent with disease severity as well as progression to deterioration or recovery^29^. Our serial observations of coagulation parameters demonstrated highly synchronous alterations of D-dimer along with progression to cardiac injury, suggesting a strong correlation between them. Interestingly, we also observed that fibrinogen was dramatically decreased, while FDP was significantly increased in cardiac injury, suggesting a hyperfibrinolytic state at the time of cardiac injury. Meanwhile, platelets were demonstrated to be reduced in cardiac injury individuals, which could be inferred that the hypercoagulable state depletes the platelets. Platelets are potentially involved in initiation of COVID-19 associated thrombosis, and may propagate coagulation such as primary and secondary haemostasis.

Coagulation abnormalities such as disseminated intravascular coagulation (DIC) and thrombotic disease manifested as elevated D-dimer and FDP have been reported to be highly prevalent in COVID-19, but these were rarely observed for other coronavirus infections^29,30^. The incidence of DIC was demonstrated to be very high (71.4%) in non-survivors^18^. Nevertheless, the pattern of DIC in COVID-19 seems to differ from DIC that occurs during sepsis since there is a more profound elevation of D-dimer as compared to thrombocytopenia in this disease. The hypercoagulable state may contribute to the formation of occlusive thrombus, which causes acute myocardial ischemia in patients with pre-exiting cardiovascular disease.

A newly proposed mechanism in the pathogenesis of COVID-19 is endothelial involvement or endotheliitis causing microcirculatory dysfunction across different organ systems^31^, which may in turn contribute to coagulation disorder. This theory was based on direct viral infection of endothelial cells and diffuse endothelial inflammation observed from histopathology examination of COVID-19 patients. Our recently published study showed that myocardial pericytes (a perivascular cell type that wrap around capillaries), which show strong ACE2 expression, may be the potential targets of SARS-CoV-2, inducing microvascular dysfunction^9^. The involvement of coronary microcirculation along with the hypercoagulable state during COVID-19 may then result in a thrombotic microangiopathy, leading to myocardial injury.

With better understanding of coagulation dysfunction in COVID-19, there has already been an increasing use of anticoagulant therapy in management of these patients. An early large observational cohort study revealed that systemic anticoagulation was associated with improved survival in hospitalized COVID-19 patients^32^. In a more recent large retrospective cohort (n = 4389), anticoagulation was associated with lower mortality and intubation among hospitalized COVID-19 patients. Compared with prophylactic anticoagulation, therapeutic anticoagulation was associated with lower mortality, although not statistically significant^33^. Several small retrospective cohort studies convinced the efficacy of anti-coagulation (e.g. heparin or enoxaparin) on COVID-19 patients with different degrees of severity^34–36^. A series of randomized controlled trials have been initiated to assess the causal effects of anticoagulation in different therapeutic regimens^37–41^, and the results will provide convinced evidences for the clinical utility of these drugs in the near future. Taken together, both microvascular endothelial injury and coagulation dysfunction might be novel potential mechanisms of cardiac injury in COVID-19, and anticoagulation treatment may play a beneficial role in the prevention of disease progression and avoidance of cardiac injury, and thus, may improve overall prognosis of patients with this disease.

Our study has several limitations. It was an observational study, and included only a limited number of cases from a single center. Most patients only had single time point measurements of cardiac injury biomarkers, and the cases in the longitudinal sub-group study were not sufficient. The clinical association studies of cardiac injury from larger multicenter cohorts are warranted to validate the direct connection between cardiac injury and coagulation. Our study identified the association of cardiac injury and coagulation biomarkers, and further histopathologic findings is needed about intravascular thrombi formation.

In summary, cardiac injury, manifested by elevation of cardiac biomarkers, is a common complication of COVID-19, and is an independent risk factor for in-hospital mortality. Old age, high leukocyte count, and high levels of AST, D-dimer and serum ferritin are significantly associated with cardiac injury. Multivariate regression analysis revealed old age and elevated D-dimer levels as being strong risk predictors of in-hospital mortality. Serial observations of coagulation parameters demonstrated highly synchronous alterations of D-dimer along with progression to cardiac injury. Therefore, the pathogenesis of cardiac injury in COVID-19 may be primarily due to coagulation dysfunction along with microvascular endothelial injury.

## Methods

### Study participants

This single-center, observational study included patients with COVID-19 who were admitted to the Jinyintan Hospital in Wuhan, China. We enrolled consecutive patients, who were diagnosed with this disease by SARS-CoV-2 viral nucleic acid test, from two patient wards of the hospital between January 1, 2020, and February 27, 2020. All patients underwent cardiac biomarker testing at least once at admission or during later hospital stay. A part of the patients in this cohort have been included in our previous study about the electrocardiographic (ECG) characteristics of COVID-19 patients with cardiac injury^42^. This study complied with the Declaration of Helsinki, and was approved by the Institutional Review Board of the Jinyintan Hospital Wuhan, China. Written informed consent was provided by patients, and informed consent statements were provided from legally authorized representatives for dead patients.

### Clinical data collection

The demographic characteristics, clinical history, laboratory findings, treatment and complications for study participants were collected from electronic medical records during the patients’ hospitalization by three investigators (J.T., W.H. and X.G.) who were blinded to the outcomes. The collected serum cardiac biomarkers included high-sensitivity troponin I (hs-TnI), myohemoglobin and creatine kinase-myocardial band (CK-MB) in all patients. Cardiac injury was defined as elevation of at least one of the three cardiac biomarkers above the 99th percentile upper reference limit. Most clinical data (including laboratory findings) were collected at the time of admission, but if a patient developed cardiac biomarker elevation during hospitalization, the clinical data were later obtained at the time of cardiac injury. Patients were categorized according to the presence or absence of cardiac injury. The presence of acute respiratory distress syndrome (ARDS) and acute kidney injury and the degree of disease severity were defined as previously reported^2^. Acute respiratory distress syndrome (ARDS) was defined according to the Berlin definition^43^. Acute kidney injury was identified according to the Kidney Disease: Improving Global Outcomes definition^44^. We defined the degree of severity of Covid-19 (severe vs. nonsevere) using the American Thoracic Society guidelines for community-acquired pneumonia^45^. The clinical outcomes were defined as hospital discharge or death (recorded by N.H. and P.Z.). At the time of hospital discharge, all patients were relieved from clinical symptoms, and had normal body temperature, significant resolution of pulmonary findings by chest radiography, and at least two consecutive negative results of SARS-CoV-2 viral nucleic acid test. The data processing and statistical analysis were performed by an independent investigator (L.C.).

### Statistical analysis

Continuous variables were presented as median (interquartile range [IQR]) values. Categorical variables were expressed as proportions. Comparisons between two groups were performed by Mann–Whitney U test for continuous variables or Fisher’s exact test for categorical variables, as appropriate. Paired Student’s t test was used to compare the serial results (from initial to cardiac injury) of cardiac biomarkers, cytokines and coagulation biomarkers. Spearman test was used for correlation analysis between cardiac biomarkers and other laboratory findings. Cox regression model was used to determine the risk factors of in-hospital mortality. The least absolute shrinkage and selection operator (LASSO) was used to determine nonzero coefficient features, which were considered as the most important factors of cardiac injury. Logistic regression model with odds ratio (OR) and 95% confidence intervals (CI) was used to determine the independent factors associated with cardiac injury, and to establish the prediction model of cardiac injury using the filtered features. Receiver operating characteristic (ROC) with area under curve (AUC) analyses were used to assess the prediction value, and Youden’s index was used to determine the cut-off values with optimal sensitivity and specificity. We used SPSS 19.0 (IBM, US) for statistical analysis, and all statistical calculations followed a two-tailed test. A *P* value less than 0.05 was considered statistically significant. Statistical diagrams were plotted by Graphpad Prism 5.0 (Graphpad Software, USA).

## Supplementary Information

Supplementary Tables

## Data Availability

The datasets used and/or analysed during the current study are available from the corresponding author on reasonable request.
