# Supplementary material for: Association of coagulation dysfunction with cardiac injury among hospitalized patients with COVID-19
Source: Sci Rep. 2021 Feb 24;11:4432. doi: 10.1038/s41598-021-83822-9 (PMC7904824; doi:10.1038/s41598-021-83822-9)
Supplement: Supplementary file 1 — Supplementary Tables [file 41598_2021_83822_MOESM1_ESM.pdf]

Supplemental Materials

Association of Coagulation Dysfunction with Cardiac Injury Among Hospitalized Patients with COVID-19

Short Title: Cardiac Injury and coagulation in COVID-19

Liang Chen, MD, PhD,[a*] Wei Hu, MD[b*], Xiaoxiao Guo, MD[b*], Ping Zhao, MS[c], Jia Tang, MD[b], Yuwei Gu, MD[b], Ninghao Huang, BS[c], Chao Wang, MS[b], An Cui, MS[b], Dian Zhang, MD[b], Linjie Hu, BS[c], Yi Feng, PhD[d], Shengshou Hu, MD[a], Mingquan Chen, MD[b#], Firat Duru, MD[e,f#], Chenglong Xiong, PhD[c,g#]

[a]State Key Laboratory of Cardiovascular Disease, Fuwai Hospital, National Center for Cardiovascular Diseases, Chinese Academy of Medical Sciences and Peking Union Medical College, Beijing, China; [b]Department of Emergency, Huashan Hospital, Fudan University, Shanghai, China; [c]Department of Epidemiology, School of Public Health, Fudan University, Shanghai, China; [d]Department of Integrative Medicine and Neurobiology, School of Basic Medical Sciences, Fudan University, Shanghai, China; [e]Department of Cardiology, University Heart Center, Zurich, Switzerland; [f]Center for Integrative Human Physiology, University of Zurich, Zurich, Switzerland; [g]Key Laboratory of Public Health Safety, Ministry of Education, School of Public Health, Fudan University, Shanghai, China;

*These authors contributed equally to this work

Prof. Shengshou Hu worked as senior author.

[#]Address for correspondence

Prof. Chenglong Xiong, 130 Dong’an Road, Shanghai 200032, China, Tel: +86 21 54230297, Fax: +86 21 54237237, xiongchenglong@fudan.edu.cn

Prof. Dr. Mingquan Chen, 12 Middle Urumqi Road, Shanghai 200040, China, Tel: +86 21 54237435, Fax: +86 21 54237435, mingquanchen@fudan.edu.cn

Prof. Dr. Firat Duru, Raemistrasse 100, Zurich CH-8091, Switzerland, Tel: +41 44 2553565, Fax: +41 44 2554401, firat.duru@usz.ch

Table S1. Clinical characteristics of COVID-19 patients (survivors and non-survivors)

Characteristics	Total (n=181)	Survivors (n=147)	Non-survivors (n=34)	*P* value
Male	102 (56.4)	81 (55.1)	21 (61.8)	0.481
Age, years	55 (46,65)	53 (43,64)	64.5 (53,71)	<0.001
Heart rate	88 (80,96)	87 (80,94)	94 (84,103)	0.033
Systolic pressure, mmHg	128 (116,142)	127 (116,141)	133.5 (115,144)	0.729
Diastolic pressure, mmHg	80 (72,85)	80 (72,85)	79 (70,85)	0.462
Signs and comorbidities, n(%)				
Fever	155 (85.6)	124 (84.4)	31 (91.2)	0.42
Cough	139 (76.8)	111 (75.5)	28 (82.4)	0.396
Chest tightness	90 (49.7)	67 (45.6)	23 (67.6)	0.021
Palpitation	4 (2.2)	3 (2)	1 (2.9)	0.568
Dyspnea	81 (44.8)	61 (41.5)	20 (58.8)	0.068
Fatigue	75 (41.4)	63 (42.9)	12 (35.3)	0.421
Sputum	51 (28.2)	36 (24.5)	15 (44.1)	0.022
Muscle ache	14 (7.7)	14 (9.5)	0 (0)	0.075
Diarrhea	16 (8.8)	13 (8.8)	3 (8.8)	1
Chest pain	5 (2.8)	4 (2.7)	1 (2.9)	1
Headache	10 (5.5)	9 (6.1)	1 (2.9)	0.69
Sore throat	10 (5.5)	8 (5.4)	2 (5.9)	1
Hypertension	54 (29.8)	42 (28.6)	12 (35.3)	0.441
Diabetes	29 (16)	25 (17)	4 (11.8)	0.454
Coronary heart disease	8 (4.4)	5 (3.4)	3 (8.8)	0.173
Arrhythmias	5 (2.8)	3 (2)	2 (5.9)	0.237
COPD	8 (4.4)	3 (2)	5 (14.7)	0.007
Cancer	7 (3.9)	4 (2.7)	3 (8.8)	0.124
Infective disease	10 (5.5)	8 (5.4)	2 (5.9)	1
Lab findings, median (IQR)				
SpO2 (%)	96 (90,98)	97 (93,98)	88 (77,94)	<0.001
Leukocytes, /μL	5.61 (4.08,8.48)	5.27 (3.87,7.48)	8.49 (5.33,11.26)	0.001
Erythrocytes, /μL	4.19 (3.86,4.56)	4.22 (3.9,4.56)	4.08 (3.69,4.59)	0.239
Hemoglobin, g/dL	129 (117,136)	129 (119,137)	118 (112,131)	0.046
Platelets, /μL	191 (147,251)	194 (153,269)	145.5 (98,224)	0.001
Lymphocytes, /μL	0.98 (0.67,1.61)	1.17 (0.8,1.72)	0.66 (0.45,0.87)	<0.001
Creatinine, μmol/L	68.4 (56.7,81.7)	68 (56.8,79.5)	70.8 (55.9,93)	0.331
eGFR, ×mL/(min×1.73m^2)	106.13 (91.02,127.69)	109.25 (93.72,126.45)	102.22 (83.01,138.85)	0.374
Albumin, g/L	33.1 (29.2,36.5)	33.5 (30.5,37.3)	28.7 (25.8,33.1)	<0.001

Total bilirubin, μmol/L	12.6 (9.4,15.9)	12.4 (9.4,15.4)	14.15 (9.4,23.4)	0.12
AST, U/L	34 (25,45)	32 (25,41)	43 (32,52)	0.004
ALT, U/L	33 (22,46)	32 (20,46)	34.5 (25,53)	0.289
Cholesterol, mmol/L	3.88 (3.19,4.53)	3.92 (3.22,4.55)	3.82 (3.07,4.36)	0.27
Triglyceride, mmol/L	1.2 (0.93,1.76)	1.2 (0.9,1.77)	1.14 (1,1.69)	0.671
LDL-C, mmol/L	2.21 (1.74,2.65)	2.22 (1.75,2.65)	2.21 (1.56,2.74)	0.812
HDL-C, mmol/L	1.03 (0.82,1.23)	1.08 (0.88,1.24)	0.82 (0.76,1.14)	0.005
LDH,U/L	270 (214,402)	248 (204,356)	421.5 (318,689)	<0.001
hs-CRP, mg/L*	25.9 (6.2,86.7)	15.1 (4.2,66.4)	82.35 (40,160)	<0.001
Procalcitonin, ng/mL	5 (5,10)	5 (5,5)	12.5 (5,36)	<0.001
D-dimer, μg/mL	0.73 (0.36,2.95)	0.58 (0.3,1.49)	6.02 (1.69,22.47)	<0.001
Serum ferritin, ng/mL†	609.88 (304.31,1217.78)	495.33 (277.37,938.18)	1398.89 (861.25,2000)	<0.001
ESR, mm/h	42 (27.3,63)	39.2 (26.8,59)	52.35 (43,73)	<0.001
Interleukin 6, pg/mL	8.65 (6.65,12.17)	8.32 (6.32,11.91)	10.19 (8.34,15.26)	0.015
hs-TnI, pg/mL	3.9 (1.2,12.5)	3.3 (1,9.4)	14.35 (6.9,185.3)	<0.001
Myohemoglobin, ng/mL	46.8 (28.2,88.25)	39.6 (26.4,75.4)	124.2 (74.4,289.9)	<0.001
BNP, pg/mL	31.1 (10.03,77.37)	31.1 (10,62.82)	85.6 (34.7,269)	<0.001
Creatine kinase, U/L	84 (55,163)	82 (51,147)	136.5 (67,349)	0.01
CK-MB, U/L	14 (10,18)	13 (10,17)	17.5 (13,24)	<0.001

ALT: alanine aminotransferase; AST: aspartate transaminase; BNP: B-type natriuretic peptide; CK-MB: creatine kinase-myocardial band; COPD: chronic obstructive pulmonary disease; eGFR: estimated glomerular filtration rate; ESR: erythrocyte sedimentation rate; HDL-C: high density lipoprotein cholesterol; hs-CRP: high-sensitivity C-reaction protein; LDH: Lactic dehydrogenase; LDL-C: low density lipoprotein cholesterol. *Extra levels of hs-CRP were shown as >160; †extra levels of serum ferritin were shown as >2000.

Table S2. Univariate and multivariate logistic regression analysis of prediction of cardiac injury among COVID-19 patients.

Variables	Univariate		Multivariate	
	OR (95%CI)	*P* value	OR (95%CI)	*P* value
Gender	2.70 (1.26,5.80)	0.011		0.455
Age	1.04 (1.01,1.07)	0.002	1.06 (1.02,1.10)	0.002
SpO2	0.94 (0.91,0.97)	<0.001		0.281
Leukocytes	1.13 (1.05,1.22)	0.002	1.080 (1.004,1.156)	0.051
Platelets	0.99 (0.99,1.00)	0.006		0.072
Lymphocytes	1.01 (0.96,1.05)	0.806		0.860
LDH	1.01 (1.00,1.01)	<0.001		0.394
hs-CRP	1.01 (1.01,1.02)	<0.001		0.776
PCT	1.00 (1.00,1.00)	0.824		0.151
D-dimer	1.13 (1.07,1.19)	<0.001	1.083 (1.030,1.139)	0.001
Serum ferritin	1.00 (1.00,1.00)	<0.001	1.002 (1.001,1.003)	<0.001
ESR	1.01 (0.99,1.02)	0.214		0.228
IL6	1.03 (0.98,1.08)	0.212		0.934
